# Comparison of liver oncogenic potential among human RAS isoforms

**DOI:** 10.18632/oncotarget.6931

**Published:** 2016-01-18

**Authors:** Sook In Chung, Hyuk Moon, Hye-Lim Ju, Dae Yeong Kim, Kyung Joo Cho, Silvia Ribback, Frank Dombrowski, Diego F. Calvisi, Simon Weonsang Ro

**Affiliations:** ^1^ Institute of Gastroenterology, Yonsei University College of Medicine, Seoul, Korea; ^2^ Liver Cirrhosis Clinical Research Center, Yonsei University College of Medicine, Seoul, Korea; ^3^ Institute of Pathology, University Medicine Greifswald, Greifswald, Germany

**Keywords:** RAS isoform, liver cancer, hydrodynamic transfection, KRAS splicing variant, P16-INK4A

## Abstract

Mutation in one of three *RAS* genes (i.e., *HRAS, KRAS*, and *NRAS*) leading to constitutive activation of RAS signaling pathways is considered a key oncogenic event in human carcinogenesis. Whether activated RAS isoforms possess different oncogenic potentials remains an unresolved question. Here, we compared oncogenic properties among RAS isoforms using liver-specific transgenesis in mice. Hydrodynamic transfection was performed using transposons expressing short hairpin RNA downregulating *p53* and an activated RAS isoform, and livers were harvested at 23 days after gene delivery. No differences were found in the hepatocarcinogenic potential among RAS isoforms, as determined by both gross examination of livers and liver weight per body weight ratio (LW/BW) of mice expressing HRAS^Q61L^, KRAS4B^G12V^ and NRAS^Q61K^. However, the tumorigenic potential differed significantly between KRAS splicing variants. The LW/BW ratio in KRAS4A^G12V^ mice was significantly lower than in KRAS4B^G12V^ mice (p < 0.001), and KRAS4A^G12V^ mice lived significantly longer than KRRAS4B^G12V^ mice (p < 0.0001). Notably, tumors from KRAS4A^G12V^ mice displayed higher expression of the p16^INK4A^ tumor suppressor when compared with KRAS4B^G12V^ tumors. Forced overexpression of p16^INK4A^ significantly reduced tumor growth in KRAS4B^G12V^ mice, suggesting that upregulation of p16^INK4A^ by KRAS4A^G12V^ presumably delays tumor development driven by the latter oncogene.

## INTRODUCTION

RAS proteins are small 21-kDa GTPases that activate a variety of key cellular processes including growth, proliferation, and migration [1–3]. In humans, three *RAS* genes encode four highly homologous RAS proteins: HRAS, NRAS, KRAS4A and KRAS4B, with the latter two resulting from alternative splicing of exon 4 of the *KRAS* gene [4–6]. All RAS isoforms are expressed almost ubiquitously and interact with the same activator and effector molecules, suggesting that they are functionally redundant [7–10]. However, it was proposed that the highly variable carboxyl-terminal of 25 amino acid residues might provide the isoforms with different biological functions [6, 10–12]. For example, Millan *et al.* reported that HRAS exhibited a stronger activation of NF-κB signaling than KRAS and NRAS in NIH3T3 cells, thus rendering them more resistant to staurosporine-induced apoptosis [13].

Activating mutations in *RAS* genes have been identified in approximately 15 - 30% of human cancers [3, 4]. These mutations, resulting in unrestrained RAS activity, lead to the sustained activation of diverse signaling pathways involved in carcinogenesis [2]. Cancer mutation databases (e.g., the COSMIC database; http://cancer.sanger.ac.uk/cosmic) show that mutation frequencies are highly biased among *RAS* genes in a given type of cancer [4, 9, 14]. For example, activating mutations in the *KRAS* gene prevail in lung, colon and pancreatic cancers, while mutations in *NRAS* and *HRAS* are rarely found in these cancer types. Likewise, activating mutations in *NRAS* are predominantly found in hematopoietic malignancies, where mutations in *HRAS* are rarely detected. Mutations in *HRAS* occur preferentially in tumors of the skin and salivary glands. In liver cancer, activating mutations in *KRAS* and *NRAS* are found considerably more frequently than in *HRAS* [4, 9, 14]. Based on the biased mutation frequencies observed among the *RAS* genes in different types of cancer, it was suggested that an intrinsic difference in the tumorigenic potential among the RAS isoforms might occur for a given type of cancer. In support of this hypothesis, Haigis *et al*. showed that a constitutively active form of KRAS is more tumorigenic than that of NRAS in mouse models of colon cancer, possibly explaining why mutations in *KRAS* are more frequently observed than *NRAS* in human colon tumors [15]. However, the apparent biases in mutation frequencies among the *RAS* genes in human cancers could be caused by factors other than differential oncogenic characteristics of RAS isoforms, such as differences in expression levels or mutation rates due to different genomic locations among the *RAS* genes [16, 17].

Here, we compared the tumorigenic potential of the four RAS isoforms in the liver using non-germline transgenic mouse models. The methodology employs a hydrodynamics-based transfection method, coupled with the *Sleeping Beauty* (SB) transposon system, which has been successfully used to generate various transgenic models for liver cancer [18, 19]. An open reading frame (ORF) encoding an activated form of each RAS isoform was placed under the same promoter and regulatory elements in the same transposon vectors to rule out differential regulation of transcription and translation. Further, transposons are randomly integrated in a chromosome of each cell, thus minimizing the locus effect [20].

## RESULTS

### Generation of transgenic models expressing constitutively active RAS isoforms

To investigate whether there is any difference in the hepatocarcinogenic potential among activated RAS isoforms, we developed transgenic mouse models expressing activated human RAS isoforms in the liver. For this purpose, we employed hydrodynamic transfection (HT) coupled with the *Sleeping Beauty* (SB) transposon system [18, 19].

**Figure 1:**
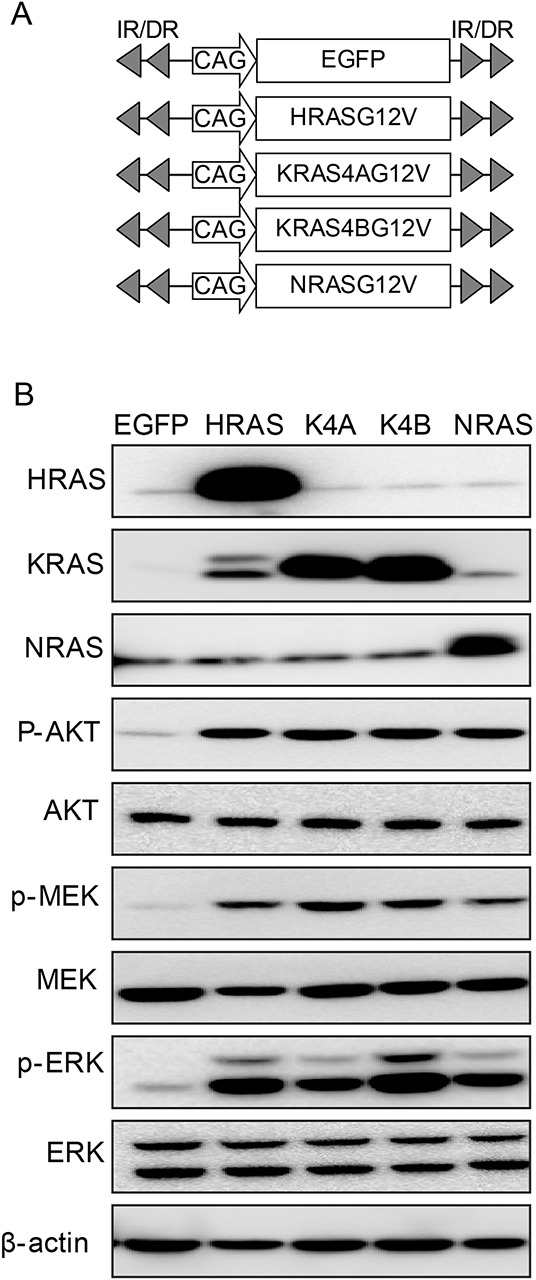
Transposons encoding each form of activated RAS **A.** Schematic illustration of transposons encoding activated RAS isoforms. Transposons encoding enhanced green fluorescent protein (EGFP) were used as a negative control. **B.** Expression of RAS and activation of downstream effector molecules were confirmed in Hep3B hepatoma cells transfected with the indicated transposons. Two days after transfection, Western blotting was performed using whole proteins extracted from cells. K4A and K4B indicate KRAS4A^G12V^ and KRAS4B^G12V^, respectively.

First, we tested the differential hepatocarcinogenic potential among RAS isoforms carrying the same activating mutation (i.e., encoding valine instead of glycine at codon 12). For this purpose, we constructed transposons encoding HRAS^G12V^, KRAS4A^G12V^, KRAS4B^G12V^, and NRAS^G12V^ by placing the open reading frame (ORF) for each activated RAS isoform into transposon-based expression vectors (Figure 1A). Isoform-specific RAS expression of the constructed transposons was confirmed by Western blotting using whole protein extracts from Hep3B cells transfected with individual transposons. Notably, Western blotting revealed that the phosphorylation levels of major downstream effectors of RAS pathways—such as AKT, MEK and ERK—were similar among cells expressing each RAS isoform (Figure 1B). Compared to cells transfected with transposons encoding enhanced green fluorescent protein (EGFP), elevated levels of the phosphorylated proteins characterized all RAS-transfected cells (Figure 1B). The results suggest that RAS proteins expressed from the transposons were equally functional.

**Figure 2:**
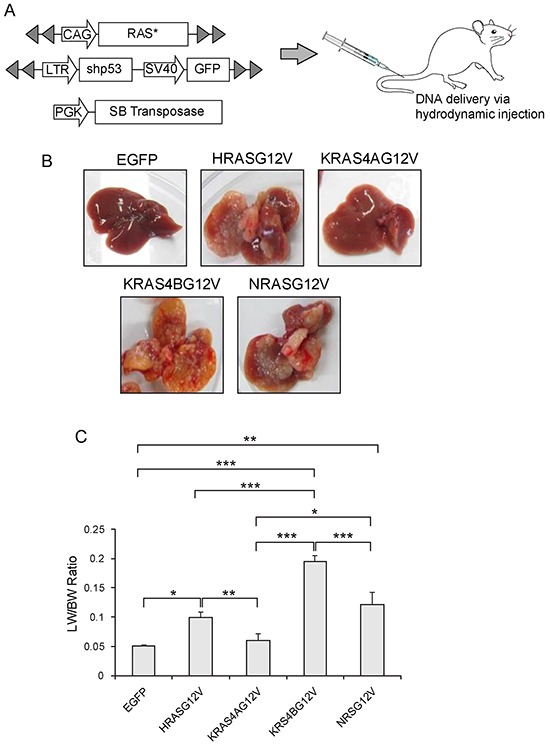
Expression of an activated RAS isoform and shp53 in the liver via hydrodynamic transfection **A.** Schematic illustration of the experimental procedure to generate transgenic livers expressing an activated form of RAS (RAS*) and short hairpin RNA downregulating *p53* (shp53). Hydrodynamic transfection was performed using a mixture of the indicated plasmids. **B.** Gross morphology of representative livers harvested at 23 days post-hydrodynamic injection from mice expressing each RAS isoform with the same activating mutation at codon 12 resulting in the substitution of valine for glycine. Liver expressing EGFP is shown as a control. **C.** Liver weight/body weight (LW/BW) ratios of mice expressing the indicated RAS isoforms. Mice expressing HRAS^G12V^, KRAS4B^G12V^ and NRAS^G12V^ had significantly higher LW/BW ratios compared to control mice expressing EGFP. Note that the LW/BW ratio of KRAS4A^G12V^ mice is similar to that of EGFP mice. The graph represents mean LW/BW ± SD. Single, double and triple asterisks indicate p < 0.05, p < 0.01 and p < 0.001, respectively.

Activated RAS alone cannot induce tumors in the liver, while concomitant *p53* knockdown using a short hairpin RNA (shp53) efficiently induces liver cancer [21]. Thus, transposons encoding each activated RAS isoform and transposons expressing shp53 were co-delivered to the liver via hydrodynamic injection along with plasmids expressing SB transposase (Figure 2A). Livers were harvested at 23 days post-hydrodynamic injection (PHI). Numerous tumor nodules were found in livers from HRAS^G12V^, KRAS4B^G12V^, and NRAS^G12V^ mice. Tumor burden was most severe in KRAS4B^G12V^ mice with tumor lesions occupying most of the liver parenchyma (Figure 2B). Much fewer and smaller nodules were detected in livers from KRAS4A^G12V^ mice compared to mice expressing other RAS isoforms. No tumors were detected in control livers expressing EGFP (Figure 2B) as well as in livers injected only with transposons encoding activated RAS (data not shown). Consistent with macroscopic observation, ratios of liver weight/body weight (LW/BW) were highest in KRAS4B^G12V^ mice and lowest in KRAS4A^G12V^ mice, in the order of KRAS4B^G12V^ > NRAS^G12V^ ≈ HRAS^G12V^ > KRAS4A^G12V^ ≈ EGFP (Figure 2C).

### Equivalent hepatocarcinogenic potential of activated HRAS, KRAS4B and NRAS genes

**Figure 3:**
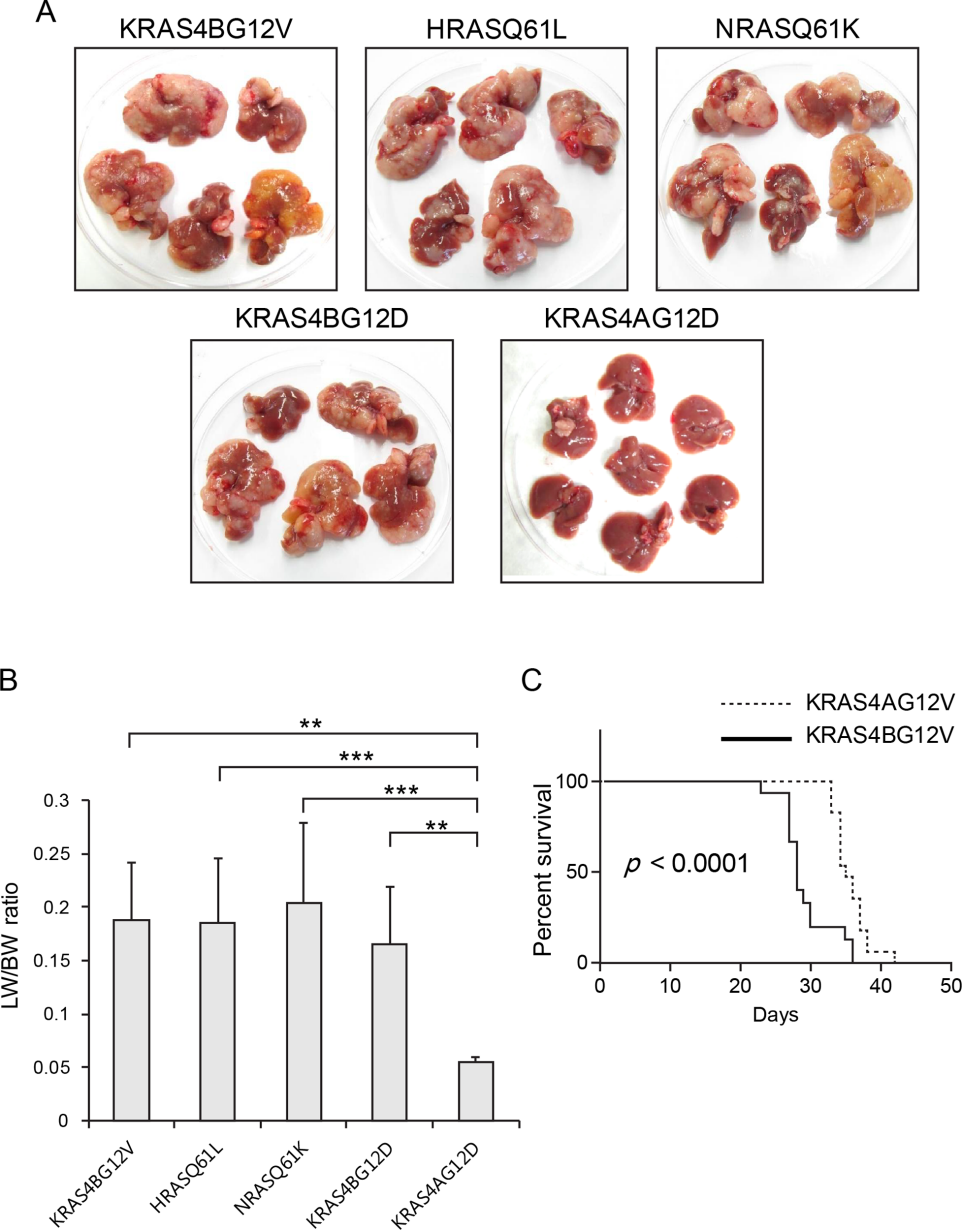
Expression of RAS isoforms with different types of activating mutations **A.** Gross morphology of representative livers harvested from mice expressing each form of activated RAS at 23 days post-hydrodynamic injection. The amino acid substitutions at codon 61 in *HRAS* and *NRAS* (resulting in HRAS^Q61L^ and NRAS^Q61K^, respectively) rendered HRAS and NRAS as oncogenic as KRAS4B^G12V^. Livers expressing KRAS4B^G12D^ had similar tumor burdens as those expressing KRAS4B^G12V^. Note that tumors were fewer and smaller in livers expressing KRAS4A^G12D^ compared to those in KRAS4B^G12D^ livers. **B.** Liver weight/body weight (LW/BW) ratios of mice expressing the indicated RAS isoforms. Note that LW/BW ratios differed significantly between KRAS4A and other RAS isoforms. The graph represents mean LW/BW ± SD. **C.** Kaplan–Meier survival curves of mice expressing KRAS4A^G12V^ and KRAS4B^G12V^ following hydrodynamic transfection. Differences in survival were highly significant (P < 0.0001).

Activating mutations can also arise in *RAS* genes at codons 13 and 61. In particular, activating mutations at codon 61 occur more frequently than at codon 12 of *HRAS* and *NRAS* in most cancer types, in contrast to *KRAS* where a mutation at codon 12 is predominant [14]. To investigate whether the tumorigenic potential can be enhanced by shifting the position of an activating mutation to codon 61 in *HRAS* and *NRAS*, transposons encoding HRAS^Q61L^ and NRAS^Q61K^ were used to induce liver tumors. When livers were harvested at 23 days PHI, no differences were detected in tumor burden among livers expressing KRAS4B^G12V^, HRAS^Q61L^ and NRAS^Q61K^ (Figure 3A). Consistently, no differences were seen in LW/BW ratios among the three groups (Figure 3B). This strongly indicates that the intrinsic oncogenic potential does not differ among *HRAS*, *NRAS* and *KRAS* proto-oncogenes and that the codon positions of an activating mutation in *RAS* can affect the oncogenic potential of a given type of RAS isoform. Although activating mutations in *HRAS* are observed considerably less frequently in liver cancer compared to *NRAS* or *KRAS* [4, 9, 14], our data show that activated HRAS has an oncogenic potential equal to that of NRAS or KRAS counterparts in the liver, calling for further research to explain the biased mutation frequencies among the RAS isoforms found in human cancers.

Another interesting question regarding mutations in *RAS* is whether activating mutations at the same codon position that result in different amino acid substitutions endow RAS with different oncogenic characteristics. Previous studies using knock-in mouse models showed that KRAS^G12D^ induced hyperplasia in tissues more efficiently than KRAS^G12V^, raising the possibility that KRAS^G12D^ and KRAS^G12V^ might have different oncogenic potentials [22, 23]. To address this question, transposons encoding KRAS^G12D^ and KRAS^G12V^ were used for hydrodynamic transfection. When livers were harvested and grossly examined at 23 days PHI, KRAS^G12D^ and KRAS^G12V^ livers showed no significant differences in tumor burden (Figure 3A). The LW/BW ratios confirmed this finding (Figure 3B). Thus, we speculate that the substitution of valine and aspartic acid at codon 12 has a similar effect on the oncogenic potential of KRAS, at least in the mouse liver.

### Activated KRAS4A is less oncogenic than activated KRAS4B in the liver

To test whether the differences in tumor growth between KRAS4A^G12V^ and KRAS4B^G12V^ livers can also be seen in another frequently found mutation in the *KRAS* gene, a hydrodynamic injection experiment was performed using transposons encoding KRAS4A^G12D^ and KRAS4B^G12D^. As found in KRAS4A^G12V^ and KRAS4B^G12V^ mice, KRAS4A^G12D^ mice had fewer and smaller tumors in the liver than KRAS4B^G12D^ mice at 23 days after hydrodynamic injection (Figure 3A). The LW/BW ratio of KRAS4A^G12D^ mice was almost identical to that of EGFP mice, and about four folds smaller than that of KRAS4B^G12D^ mice (p < 0.01; see Figure 3B).

The results suggest that KRAS4A is less oncogenic than KRAS4B in the liver, irrespective of the type of activating mutation. In addition, the present data suggest that the KRAS4B splicing variant is the major driver of hepatocarcinogenesis when an activating mutation occurs in the *KRAS* gene. In accordance with these findings, it has been reported that the KRAS4A/4B transcript ratio is significantly reduced in colorectal tumors when compared with that in corresponding non-tumor tissues, further suggesting that activated KRAS4B might be more oncogenic than activated KRAS4A in cancer [24, 25].

To evaluate the effect of reduced tumor growth in KRAS4A^G12V^ livers on the survival of mice, we compared the survival of KRAS4A^G12V^ mice (n=17) and KRAS4B^G12V^ (n=15) mice following hydrodynamic transfection. The survival analysis showed that KRAS4A^G12V^ mice survived significantly longer than KRAS4B^G12V^ mice (p < 10^−4^), suggesting that reduced tumor development in KRAS4A^G12V^ livers prolonged their life span (Figure 3C).

### Reduced cellular proliferation in KRAS4A^G12V^ tumors

**Figure 4:**
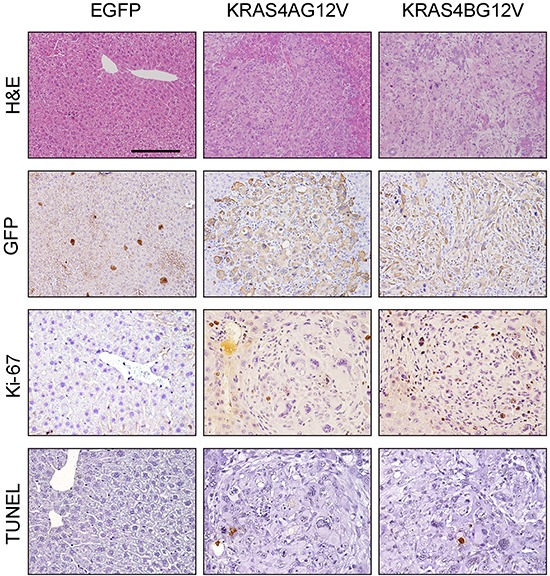
Histological analysis of tumors expressing KRAS4A^G12V^ and KRAS4B^G12V^ Paraffin sections of KRAS4A^G12V^ and KRAS4B^G12V^ tumors, as well as liver parenchyma from EGFP mice, were stained with H&E, anti-GFP and anti-Ki-67 antibodies and TUNEL reagents. Scale bar: 100 μm for H&E and GFP staining and 50 μm for Ki-67 and TUNEL staining.

Histopathologic examination showed that tumors from both KRAS4A^G12V^ and KRAS4B^G12V^ groups were poorly differentiated HCC. There were no phenotypic differences in tumor cells between the two groups (Figure 4). Tumor cells revealed an almost identical morphology of highly atypical nuclei. Immunohistochemical (IHC) analysis with antibodies against GFP confirmed that the tumors in both groups originated from cells transfected with the transposons (Figure 4).

To characterize the reduced tumor growth in KRAS4A^G12V^ mice at the cellular level, cell proliferation and apoptosis were investigated in the tumor. Proliferation of tumor cells was significantly reduced in KRAS4A^G12V^ compared to KRAS4B^G12V^, as determined by Ki-67 staining (Figure 4). Apoptotic cells were rarely detected in tumor sections from both groups using terminal deoxynucleotidyltransferase-mediated dUTP-biotin nick-end labeling (TUNEL) staining (Figure 4). Thus, the decreased level of cellular proliferation is likely responsible for the slower growth of tumors expressing KRAS4A^G12V^.

### Upregulation of p16^INK4A^ in tumors of KRAS4A^G12V^

**Figure 5:**
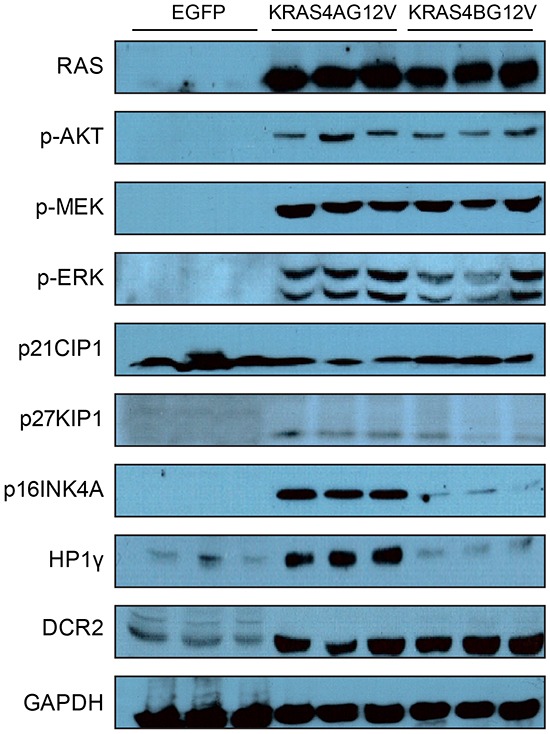
Expression levels of various genes and phosphorylation levels of RAS downstream effector molecules Tumors were harvested from mice and whole proteins were extracted from each tumor. Protein extracts from livers transfected with EGFP were used as controls. Expression levels of the indicated genes and phosphorylation levels of the indicated proteins were assessed by immunoblotting using the appropriate antibodies. Note that the expression level of P16^INK4A^, a cyclin-dependent kinase inhibitor, was significantly upregulated in KRAS4A^G12V^ tumors compared to KRAS4B^G12V^ tumors.

To characterize the reduced cellular proliferation in KRAS4A^G12V^ tumors at the molecular level, some players in RAS-dependent growth were compared between KRAS4A^G12V^ and KRAS4B^G12V^ tumors via Western blotting. Whole proteins were extracted from KRAS4B^G12V^ tumors at 23 days PHI and used for immunoblotting. Because nodules were too small in KRAS4A^G12V^ livers at 23 days PHI, whole protein extracts from KRAS4A^G12V^ tumors at 37 days PHI were used instead for this study. Western blotting showed that expression levels of RAS were similar between KRAS4A^G12V^ and KRAS4B^G12V^ tumors (Figure 5). Major downstream effector molecules of RAS signaling pathways were also similarly activated in both groups, as determined by phosphorylation levels of AKT, MEK, and ERK (Figure 5). Thus, the reduced tumor growth of KRAS4A^G12V^ tumors does not depend on low level of the transgene expression or insufficient activation of the PI3K-AKT and RAF-MEK-ERK downstream pathways by the activated KRAS protein.

**Figure 6:**
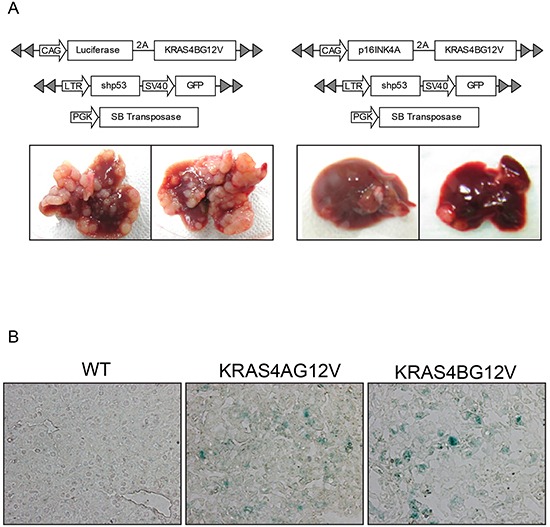
Overexpression of P16^INK4A^ suppresses tumor growth driven by activated KRAS **A.** Transposons were constructed that co-express KRAS4B^G12V^ and p16^INK4A^ (or firefly luciferase as a control) in a single open reading frame via 2A-mediated ribosome skipping. Hydrodynamic transfection was performed using the indicated plasmids. Gross morphology of representative livers is shown below. **B.** Frozen sections of KRAS4A^G12V^ and KRAS4B^G12V^ tumors were assayed using senescence-associated β-gal staining. No difference in β-gal staining level was observed between tumors expressing KRAS4A^G12V^ and KRAS4B^G12V^.

Because reduced cellular proliferation was observed in KRAS4A^G12V^ mice, expression levels of genes involved in cell cycle regulation were assessed. We were particularly interested in cyclin-dependent kinase inhibitors (CDKIs) suppressing cell cycle progression because RAS can activate various CDKIs, such as p16^INK4A^ and p21^Cip1^ [26, 27]. Western blotting revealed that the level of p16^INK4A^ was significantly higher in KRAS4A^G12V^ tumors compared to KRAS4B^G12V^ tumors, while p21^Cip1^ and P27^Kip1^ expression did not differ between KRAS4A^G12V^ and KRAS4B^G12V^ malignant lesions (Figure 5). To investigate whether upregulation of p16^INK4A^ can suppress KRAS-driven tumorigenesis, transposons were constructed in which p16^INK4A^ was co-expressed with KRAS4B^G12V^ (Figure 6A). Compared to the control gene encoding firefly luciferase, overexpression of p16^INK4A^ significantly suppressed hepatocarcinogenesis induced by KRAS4B^G12V^ (Figure 6A). Although further analysis is required to elucidate the underlying molecular mechanism, the findings suggest that an activated KRAS4A potentiates upregulation of p16^INK4A^, which, in turn, functions as a critical tumor suppressor in KRAS4A-driven hepatocarcinogenesis [28].

Upregulated p16^INK4A^ can induce senescence in cells expressing an activated RAS *in vitro* and *in vivo*, known as oncogene-induced senescence (OIS) [26, 29, 30]. To investigate whether senescence was involved in p16^INK4A^ -mediated tumor suppression in KRAS4A^G12V^ tumors, Western blotting was performed using antibodies against heterochromatin protein 1 gamma (HP1γ) and decoy receptor 2 (DCR2), molecular markers for senescence [31]. Although the expression level of HP1γ was higher in KRAS4A^G12V^ tumors than in KRAS4B^G12V^ tumors, no difference was seen in DCR2 levels between the two groups (Figure 5). Further, a senescence-associated β-gal assay showed no staining difference in KRAS4A^G12V^ and KRAS4B^G12V^ tumors, suggesting that tumor suppression in KRAS4A^G12V^ tumors by upregulated p16^INK4A^ is not mediated by cellular senescence (Figure 6B).

**Figure 7:**
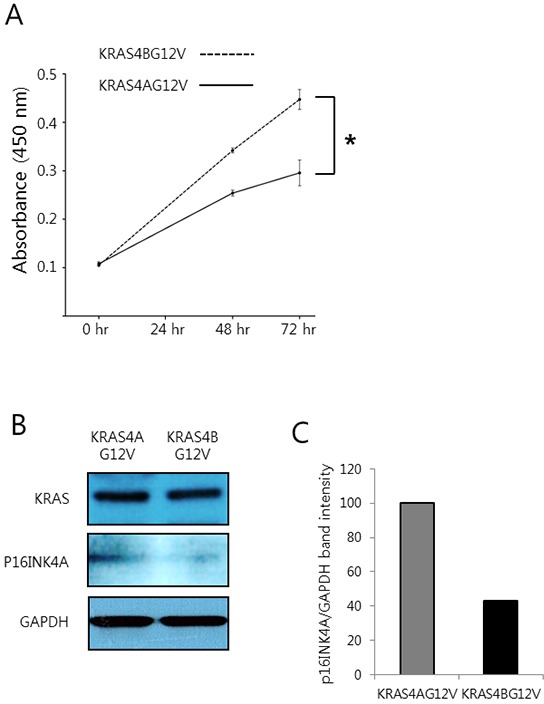
Colorectal cancer cells expressing KRAS4B^G12V^ are more proliferative than those expressing KRAS4A^G12V^ **A.** An MTT assay was performed at the indicated time points using the SW48 human colorectal cancer cell line stably expressing KRAS4A^G12V^ and KRAS4B^G12V^. An asterisk (*) indicates *p* < 0.05. **B.** Western blotting was performed using whole proteins extracted from the colorectal cancer cells stably expressing KRAS4A^G12V^ and KRAS4B^G12V^. **C.** Densitometry analysis of the western blots shown in (B). Values represent the relative ratio of p16^INK4A^ /GAPDH in cells expressing KRAS4A^G12V^ and KRAS4B^G12V^.

Since oncogenic mutations in *KRAS* are predominantly found in human lung, pancreatic and colorectal tumors, it would be intriguing to assess whether KRAS4A and 4B would display a differential carcinogenic potential in those tissues as well. However, the *in vivo* evaluation of KRAS4A and KRAS4B oncogenic potential in extrahepatic tissues is not as simple as in the liver due to the lack of an efficient and simple method for tissue-specific transgenesis such as hydrodynamic transfection. As an alternative approach, we investigated the oncogenic potential of KRAS4A and 4B using the SW48 human colorectal cancer cell line, which harbors no mutation in *RAS* genes [32]. SW48 cells stably expressing KRAS4B^G12V^ grew significantly faster than those expressing KRAS4A^G12V^ (Figure 7A), suggesting that KRAS4B is likely more oncogenic than KRAS4A in colorectal tissue. A higher expression of p16^INK4A^ was observed in colorectal cancer cells expressing KRAS4A^G12V^, compared to those expressing KRAS4B^G12V^, although both cells exhibited similar levels of KRAS (Figure 7B and 7C).

## DISCUSSION

In this study, we compared the oncogenic potential among activated RAS isoforms in the mouse liver and found that no differences in intrinsic oncogenic potential among *HRAS*, *KRAS* and *NRAS* exist. One can assume differential oncogenic characteristics among RAS isoforms from a high frequency of activating mutations in a specific *RAS* gene for a given type of cancer [4, 9, 14]. However, other factors could result in biased frequencies of activated RAS isoforms in cancers, including differences in tissue-specific gene expression levels and/or mutation rates among the RAS genes due to their different genomic locations. In line with our findings, To *et al*. showed that *HRAS* located at the endogenous genomic location of *KRAS* underwent an activating mutation and induced lung tumors as efficiently as *KRAS* [33].

The oncogenic potential differed significantly between the two KRAS splicing variants, with KRAS4B being more tumorigenic than KRAS4A in the liver. Thus, it is presumed that when an activating mutation arises in *KRAS,* KRAS4B will predominantly lead the tumorigenic processes. In colorectal cancer, it was reported that the KRAS4B isoform is selectively overexpressed and thus, the ratio of KRAS4A/4B isoforms is reduced during colorectal carcinogenesis, suggesting that KRAS4B might be more tumorigenic than KRAS4A [24, 25].

Although the mechanism underlying the reduced oncogenicity by KRAS4A was not fully investigated in this study, we detected a significant upregulation of p16^INK4A^ in KRAS4A-driven liver tumors compared to KRAS4B tumors. Ectopic expression of p16^INK4A^ led to significantly reduced tumor growth induced by activated KRAS4B^G12V^ (Figure 6A), emphasizing the tumor-suppressive role of upregulated p16^INK4A^ in KRAS-driven hepatocarcinogenesis. p16^INK4A^ can prevent cell proliferation by inhibiting cell cycle progression. We detected a decreased level of cell proliferation in KRAS4A tumors compared to KRAS4B tumors, but no differences were seen in apoptosis and senescence levels between the two tumors (Figures 4 and 6B). Further investigation is needed to understand the mechanism whereby activated KRAS4A upregulates p16^INK4A^ to a greater degree than activated KRAS4B.

## MATERIALS AND METHODS

### Plasmids

The pT2/shp53/GFP4, pT2/EGFP, and pPGK-SB13 plasmids were described previously [21].

Human cDNA encoding HRAS, KRAS4B and NRAS were used for site-directed mutagenesis to generate open reading frames (ORFs) encoding HRAS^G12V^, HRAS^Q61L^, KRAS4B^G12V^, KRAS4B^G12D^, NRAS^G12V^, and NRAS^Q61K^. ORFs encoding KRAS4A^G12V^ and KRAS4A^G12D^ were generated from KRAS4B^G12V^ and KRAS4B^G12D^, respectively via PCR using the following primer pairs: forward, 5′-ATG ACT GAA TAT AAA CTT GTG GTA GTT-3′; and reverse, 5′-TTA CAT TAT AAT GCA TTT TTT AAT TTT CAC ACA GCC AGG AGT CTT TTC TTC TTT GCT GAT TTT TTT CAA TCT GTA TTG TCG GAT CTC CCT CAC CAA TGT ATA AAA AGC ATC CTC CAC TCT CTG TCT TGT CTT TGC TGA TGT TTC-3′. Each ORF encoding an activated RAS was substituted for EGFP cDNA in pT2/EGFP. Transposons co-expressing p16^INK4A^ and KRAS4B^G12V^ were generated as follows. After removing the termination codon in cDNA encoding p16^INK4A^, the DNA sequence encoding the *Thosea asigna* virus (TaV) 2A peptide with a GSG linker at the N-terminus (i.e., GSGEGRGSLLTCGDVEENPGP) was placed, in-frame, between cDNA encoding p16^INK4A^ and cDNA encoding KRAS4B^G12V^ [34]. Subsequently, the fusion DNA was inserted in pT2/EGFP after removing EGFP cDNA. Transposons co-expressing firefly luciferase and KRAS4B^G12V^ were constructed in the same manner.

### Transfection and western blotting

Hep3B cells were transfected with 2μg of plasmid DNA using FuGENE HD Transfection Reagent (Promega, Madison, WI, USA), according to the manufacturer's instructions. Two days post transfection, cells were lysed in 1× RIPA buffer (#9806; Cell Signaling Technology, Danvers, MA, USA). Western blot experiments were performed using standard methods. Anti-HRAS (sc-520; Santa Cruz Biotechnology, Santa Cruz, CA, USA), anti-KRAS (sc-30; Santa Cruz Biotechnology), anti-NRAS (sc-519; Santa Cruz Biotechnology), anti-AKT (#9272, Cell Signaling Technology, Danvers, MA, USA), anti-phospho-AKT (#4060, Cell Signaling Technology), anti-MEK (#9126, Cell Signaling Technology), anti-phospho-MEK (#9154, Cell Signaling Technology), anti-ERK (#9102, Cell Signaling Technology), anti-phospho-ERK (#4370, Cell Signaling Technology), anti-p16^INK4A^ (10883-1-AP; Proteintech, Chicago, IL, USA), anti-GAPDH (#2118; Cell Signaling Technology), and anti-β-actin (sc-47778; Santa Cruz Biotechnology) were used as primary antibodies, and horseradish peroxidase (HRP)-conjugated anti-rabbit IgG (A0545;Sigma-Aldrich, St. Louis, MO, USA) was used as the secondary antibody.

### Stable cell lines and cell proliferation assay

The human SW48 colorectal cancer cell line was transfected with 2μg of pcDNA3- KRAS4A^G12V^ or KRAS4B^G12V^. Two days post transfection, cells were placed in culture media containing G418 disulfate salt (G-8168; Sigma-Aldrich) for the selection of stably expressing cells. To evaluate proliferation, colorectal cancer cells stably expressing KRAS4A^G12V^ and KRAS4B^G12V^, respectively, were seeded at a density of 1,500 cells per well in a 96-well plate. At specified time points, an 3-(4,5-dimethylthiazol-2-yl)-2,5-diphenyltetrazolium bromide (MTT) assay was performed according to the manufacturer's instructions (Cell Viability, Proliferation and Cytotoxicity Assay kit; DOGEN, Seoul, Korea). Absorbance at 450 nm was measured using a microplate reader (VersaMax ELISA Microplate Reader, Molecular Devices, Sunnyvale, CA, USA).

### Animal experiments

All experiments using live mice were approved by the Animal Policy and Welfare Committee of the Yonsei University College of Medicine (Seoul, Korea). Wild-type C57BL/6 mice were purchased from Orientbio (Seongnam, Korea). For hydrodynamic injection, 12.5 μg of transposons encoding an activated RAS were mixed with 14 μg of pT2/shp53/GFP4 and 9 μg of pPGK-SB13, and then suspended in 2 ml of Lactated Ringer's solution. Each DNA solution was injected into the lateral tail vein of 6-week-old C57BL/6 male mice (0.1 ml/g body weight) in less than 7 seconds.

### Liver harvesting and histopathological analysis

Mice were deeply anesthetized by intraperitoneal injection of zoletil (30 mg/kg) and xylazine (10 mg/kg). A midline laparotomy incision was performed, and their livers were removed and fixed overnight in freshly prepared 10% neutral-buffered formalin. The remainder of the liver was snap-frozen in liquid nitrogen and stored at −70°C until use. Fixed tissue samples were embedded in paraffin and sectioned into 4-μm slices. Slices were stained with hematoxylin & eosin (H&E). Liver lesions were assessed by certified pathologists and liver experts (S.R. and F.D.) in accordance with the criteria established by Frith et al [35].

### Immunohistochemistry (IHC) and TUNEL assay

Paraffin sections were deparaffinized in xylene and rehydrated through a gradual decrease in ethanol concentration. Antigen epitopes were then unmasked using sodium citrate buffer (pH 6.0). Subsequently, the sections were incubated overnight at 4°C using the following primary antibodies: anti-GFP (#2555; Cell Signaling Technology), anti-Ki-67 (ab15580; Abcam, Cambridge, UK). After primary antibody incubation, sections were incubated with a biotinylated anti-rabbit IgG secondary antibody (PK-7200; Vector Laboratories, Burlingame, CA, USA) followed by treatment with freshly prepared DAB substrates (PK-4100; Vector Laboratories). Sections were lightly counter-stained with hematoxylin and mounted. Apoptosis was assessed in liver sections using terminal deoxynucleotidyl transferase-mediated dUTP-biotin nick end-labeling (TUNEL) staining (ApopTag® *In Situ* Apoptosis Detection Kits; Merck, Billerica, MA, USA). Slides were analyzed and photographed using a microscope (Eclipse Ti; Nikon, Tokyo, Japan) equipped with a digital camera.

### Protein harvest from liver and western blotting

Liver tissues were homogenized and digested in 1× RIPA buffer containing phosphatase inhibitor cocktail solution (GenDEPOT, Barker, TX, USA). Western blot experiments were performed following the standard protocol. The following primary antibodies were used: anti-Pan-RAS (sc-14022; Santa Cruz Biotechnology), anti-phospho-AKT (#4060, Cell Signaling Technology), anti-phospho-MEK (#9154, Cell Signaling Technology), anti-phospho-ERK (#4370, Cell Signaling Technology), anti-p21^Cip1^ (ab2961; Abcam), anti-p27^Kip1^ (ab7961; Abcam), anti-p16^INK4A^ (10883-1-AP; Proteintech), anti-HP1γ (ab10480, Abcam), anti-DcR2 (ab2019; Abcam), and anti-GAPDH (#2118; Cell Signaling Technology). Anti-rabbit IgG–HRP (Sigma-Aldrich) was used as the secondary antibody. Bands were detected using the enhanced chemiluminescence (ECL) Western blot detection system (Amersham Pharmacia Biotech, Piscataway, NJ, USA).

### Senescence-associated β-galactosidase (SA-β-gal) assay

Frozen tissues embedded in OCT compound were sectioned into 6 μm slices. Sections were immediately immersed in a fixative solution (2% formaldehyde and 0.2% glutaraldehyde in PBS) for 15 min at room temperature. Following fixation, sections were stained with β-gal staining solution following the manufacturer's instructions (#9860; Senescence β-galactosidase staining kit; Cell Signaling Technology). After overnight staining, the β-gal-stained sections were analyzed using a microscope (Eclipse Ti; Nikon).

### Statistical analysis

The liver weight/body weight ratio (LW/BW) data were expressed as the means ± SD with sample sizes n = 5 or larger. Statistical analyses of these data were conducted via an unpaired parametric Student's t-test. Significant differences between two groups were denoted by asterisks (*, *p* < 0.05; **, *p* < 0.01; ***, *p* < 0.001). Kaplan–Meier survival data were evaluated using a log-rank test.
